# Adrenoceptor stimulation does not affect ICAM-1 and VCAM-1 expression *in vitro*

**DOI:** 10.1186/1756-0500-4-40

**Published:** 2011-02-25

**Authors:** Heiner Ruschulte, Dirk Scheinichen, Martijn van Griensven, Susanne Weyrauch, Wibke Liefing, Birgitt Harrmeijer, Michael Przemeck, Björn Jüttner

**Affiliations:** 1 Dept. of Anaesthesiology & Intensive Care Medicine, Hannover Medical School Hannover, Germany; 2 Department of Trauma Surgery, Hannover Medical School Hannover, Germany

## Abstract

**Background:**

Endothelial adhesion molecules ICAM-1 (CD54) and VCAM-1 (CD106) mediate cellular adhesion and transcellular migration. Cell adhesion and diapedesis have a key role in the course of shock and sepsis. During severe sepsis, adrenoceptor agonist levels may be increased due to endogenous production or due to intensive care treatment. As yet, the influence of β1 or β2 agonists on adhesion molecule formation on endothelial cells has remained unclear.

**Methods:**

Cultured human umbilical vein endothelial cells were stimulated with *E. coli*. Following bacterial stimulation the cells were incubated with either β2 receptor agonist terbutaline or β1 agonist norepinephrine. ICAM-1 and VCAM-1 expression were examined using flow cytometry.

**Results:**

Administration of norepinephrine did not cause increases of both CD54 and CD106 in stimulated HUVEC. Compared to negative controls the bacterial stimulation itself led to an increase of adhesion molecules. Following administration of terbutaline no significant increase in CD54 expression was found.

**Conclusions:**

Bacterial stimulation led to an increase of adhesion molecule expression. Adrenoceptor stimulation of activated endothelial cells did not cause significant increases of cellular adhesion molecules.

## Background

Endothelial cells are found in every vascularized tissue, and form the inner lining of the vascular and capillary wall. The main function of endothelial cells is to work as a communicative barrier between intravascular and extravascular spaces. Under physiologic conditions, there are regular intercellular and transcellular routes of transport of corpuscular and noncorpuscular agents from the luminal site to the abluminal site [1]. Contact and communication among cells either from identical or different types and sites depend on receptors and adhesion molecules anchored in the membrane of the cell.

Adhesion molecules are mediators between cells. During inflammation, ELAM-1 (E-selectin), intercellular adhesion molecule 1 (ICAM-1 (CD54)) and vascular adhesion molecule 1 (VCAM-1 (CD106)) are important for the interaction with peripheral blood cells. After bacterial invasion, cytokines like IL-1β or TNF-alpha, as produced in tissue macrophages, cause E-selectin expression on endothelial surfaces. Following own selectin expression, white blood cells start rolling on endothelial surface which means deceleration and initiation of adherence. On the endothelial surface, ICAM-1 interacts with MAC-1 (= β-integrin) surface molecule of leukocytes. Contacts amongst endothelial cells and to monocytes or lymphocytes are mediated via endothelial VCAM-1 (CD106) and leukocytes surface molecule VLA-4. Upon activation, these adhesion molecules can be sledded from the cell surface and released into circulation [2].

During sepsis and multiple trauma physiological homeostasis mechanisms are disturbed. Patients frequently need pharmaceutical or mechanical organ function support. Hypodynamic or hyperdynamic circulatory dysregulation frequently require adrenoceptor agonist therapy. Under stimulation of β2 adrenoceptors, reduced adherence of NK-cells [3], increased vasorelaxation [4], and reduced extravasation of fluids were observed. Besides treating the underlying condition, microvascular resuscitation is a crucial part of sepsis treatment [5]. It was our goal to investigate whether the expression of adhesion molecules is influenced by the use of norepinephrine or terbutaline, as this may be detrimental or beneficial during sepsis. We therefore examined the *ex vivo *effects of β1 or β2 stimulation on adhesion molecule expression in human endothelial cells by flow cytometry.

The questions especially addressed were: Is there an altered expression of adhesion molecules ICAM-1 or VCAM-1 by administration of a bacterial stimulus, or by the adrenoceptor agonist, or by both?

## Methods

### Cell preparation and cell culture

Cells were harvested using 0.04% collagenase (in PBS/MgCl_2 _solution) from human umbilical cords. After centrifugation the cell pellets were put into 5 ml medium (Endothelmedium, PromoCell, Heidelberg, Germany), given into culture flasks and incubated at 37°C and 5% CO_2 _(Incubator: Queue, Nunc, Wiesbaden, Germany). Cells were trypsinized and re-seeded at roughly 90% confluence. After the second or third passage, cells were used for stimulation and investigation of both β1 agonist and β2 agonist effects.

### Experimental setting

Cells were stimulated overnight for 16 to 20 hours in the incubator (37°C, 5% CO_2_) by adding *E. coli *(3 × 10^7 ^× ml ^-1^) (H 101, grown in Hannover Medical School's own laboratory).

After washing the cells with Ly-PBS (Hannover Medical School), 50 μl trypsin EDTA (Biochrom, Berlin, Germany) were given for detachment of the cells. Then, the cells were washed twice. Finally, 5 μl of norepinephrine (Sigma Aldrich, Taufkirchen, Germany) or 10 μl terbutaline (Sigma Aldrich, Taufkirchen, Germany) in 500 μl Ly-PBS buffer were administered both at concentrations of 10^-7 ^M at a pH of 7.4. After 20 minutes' the incubations were stopped. 10 μl of each antibody (ICAM-1, i.e. CD54, Beckman Coulter, Krefeld, Germany; VCAM-1, i.e. CD106 Serotec, Düsseldorf, Germany) both marked with the green fluorescent fluorescein isothiocyanate (FITC) were added. 20 μl of the red fluorescent propidium iodide (PI; Sigma Aldrich, Taufkirchen, Germany) to stain DNA of dead cells, which should be excluded from our calculation, were also added. After 30 minutes of incubation (room temperature, dark) each tube again was centrifuged (4°C, 2000 rpm, 5 min) and the supernatant decanted to remove eventual detached adhesion molecules and antibodies. Cells were resuspended in 750 μl binding buffer solution, put into FACS tubes (Sarstedt, Nürnberg, Germany) on ice and then analysed flowcytometrically (EPICS XL, Beckman Coulter, Krefeld, Germany).

### FACS analysis

10,000 events per assay were measured after PI positive, i.e. dead, cells had been excluded. Events were depicted in dot plot diagrams to analyse the number of all events as a qualitative parameter. A life gate was then drawn around the HUVEC population. In a histogram the mean fluorescence of events as a quantitative parameter to analyse the distribution of a marked characteristic was registered.

### Data collection and statistical analysis

All measurements were collected in an Excel (Microsoft) database and analysed with SPSS (11.0), p < 0.05 was considered significant. All numeric data showed a Gaussian distribution (Kolmogorov-Smirnov-Test), so the univariate analysis of variance (ANOVA) (Fischer's exact test) was used to compare various concentrations of either β1 or β2 agonistic substances. Because not all the measurement showed skewness |γ| ≤ 0.4, differences in adhesion molecule expression were compared in a paired Wilcoxon test. Correlation of quality (percent stimulation) and quantity (mean fluorescence) was calculated with correlation coefficient (Pearson).

Results are presented as mean and standard error of mean (mean ± SEM percentage). Results were depicted as histograms reproducing the mean fluorescence which means quantitative adhesion molecule expression. We also calculated the relationships of norepinephrine administration compared to the baseline values which were depicted as box plots.

## Results

HUVEC were investigated if there was a change in expression of the cellular adhesion molecules ICAM-1 and VCAM-1 caused by either the β1-stimulating agent norepinephrine or the β2-agonist terbutaline with and without bacterial stimulation with *E. coli*.

**Figure 1:**
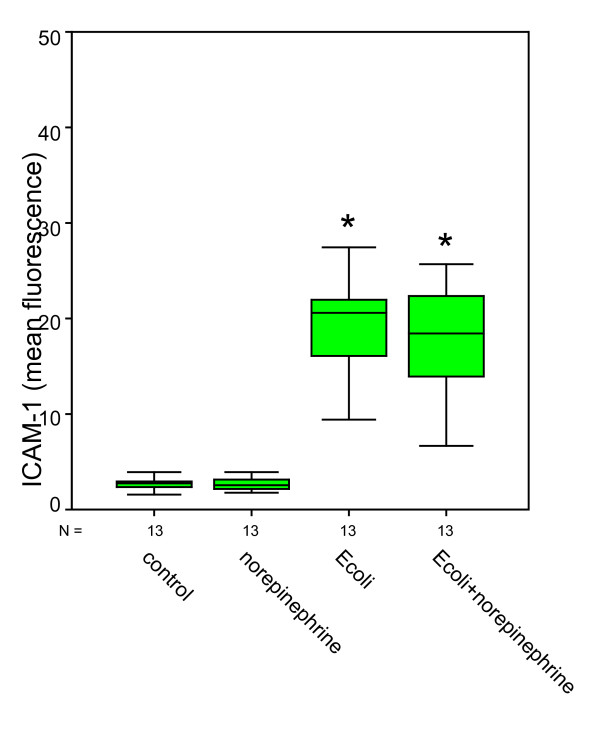
**Quantitative changes in CD 54 (ICAM-1) expression following norepinephrine (β1) and bacterial (E. coli) stimulation**. *statistical analysis p = 0.001, versus negative control and versus β1 stimulation, Wilcoxon test. (The box represents the mid 50% range of the data. Whiskers are the lines that extend outwards from the ends of the box to a distance of at most 1.5 units of interquartile range.)

**Figure 2:**
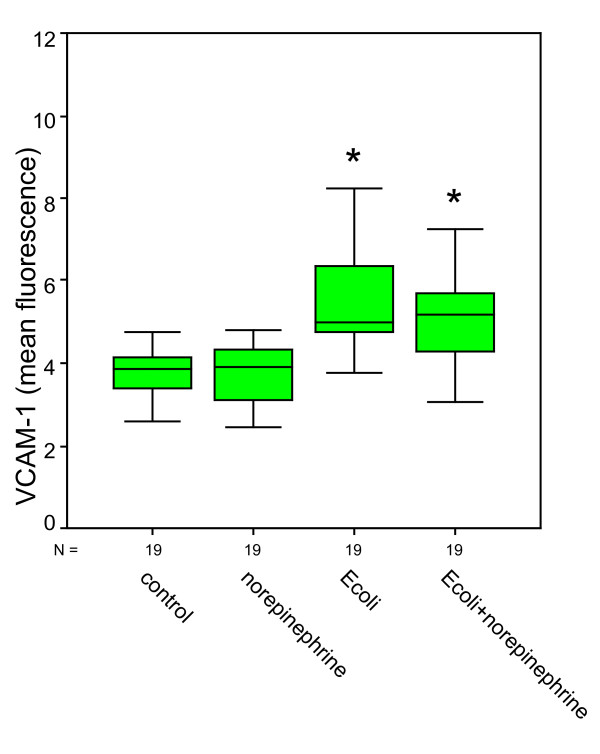
**Quantitative changes in CD 106 (VCAM-1) expression following norepinephrine (β1) and bacterial (E.coli) stimulation**. *statistical analysis p ≤ 0.001, versus negative control and versus β1 stimulation, Wilcoxon test. (The box represents the mid 50% range of the data. Whiskers are the lines that extend outwards from the ends of the box to a distance of at most 1.5 units of interquartile range.)

**Figure 3:**
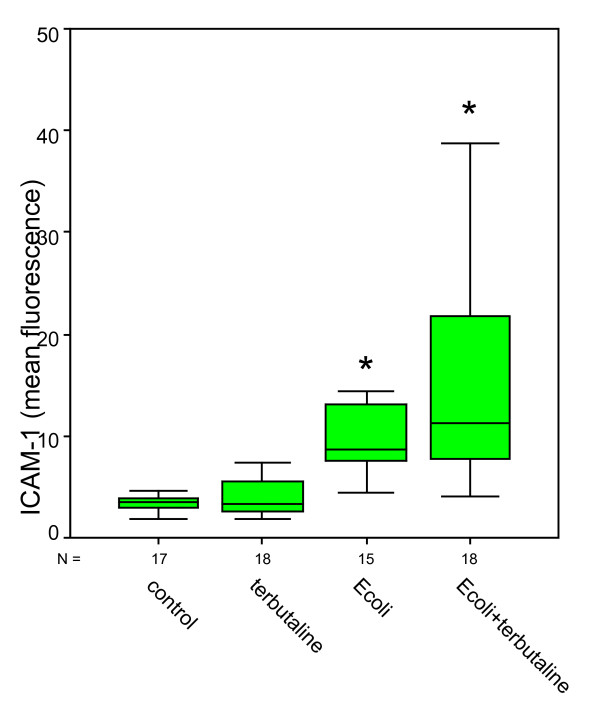
**Quantitative changes in CD 54 (ICAM-1) expression following terbutaline (β2) and bacterial (E.coli) stimulation**. *statistical analysis p ≤ 0.001, versus negative control and versus β2 stimulation, Wilcoxon test. (The box represents the mid 50% range of the data. Whiskers are the lines that extend outwards from the ends of the box to a distance of at most 1.5 units of interquartile range.)

**Figure 4:**
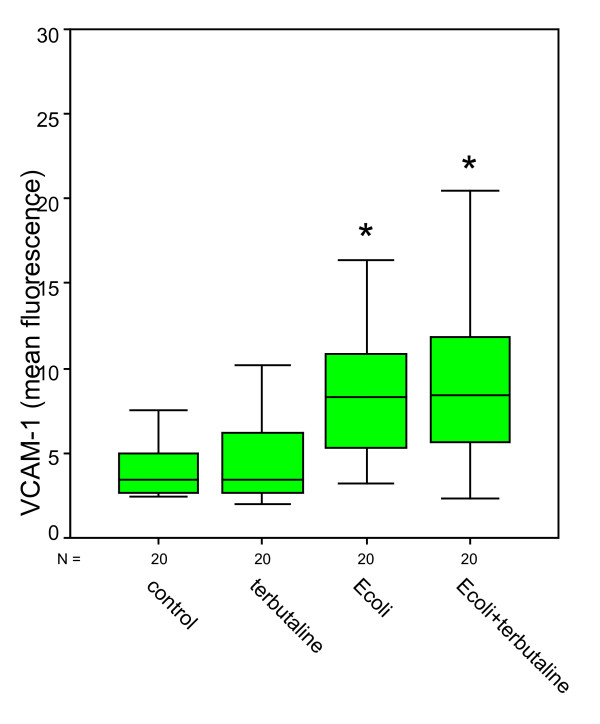
**Quantitative changes in CD 106 (VCAM-1) expression following terbutaline (β2) and bacterial (E.coli) stimulation**. *statistical analysis p ≤ 0.001, versus negative control and versus β2 stimulation, Wilcoxon test. (The box represents the mid 50% range of the data. Whiskers are the lines that extend outwards from the ends of the box to a distance of at most 1.5 units of interquartile range.)

Compared to negative controls (ICAM-1: 2.74 ± 0.18; VCAM-1: 3.76 ± 0.13), bacterial stimulation of HUVEC and additional norepinephrine administration caused a significant increase in ICAM-1 expression up to 20.64 ± 2.88 and also a significant increase in VCAM-1 expression up to 5.73 ± 0.41 (Figures 1 &2). Norepinephrine administration without bacterial stimulation did not cause relevant increases in adhesion molecule expression (ICAM-1: 2.76 ± 0.67; VCAM-1: 3.72 ± 0.18). Norepinephrine administration onto the stimulated cells caused significant effects in ICAM-1 expressions compared to negative controls, but roughly remained at levels as caused by bacterial stimulation only (ICAM-1: 19.81 ± 2.53; VCAM-1: 5.30 ± 0.37). Administration of terbutaline (Figures 3 &4) did not cause increases in both adhesion molecules investigated (negative controls 4.07 ± 0.46 in ICAM-1, and 4.42 ± 0.59 in VCAM-1, terbutaline administration 4.63 ± 0.73 in ICAM-1, and 4.80 ± 0.71 in VCAM-1, respectively). Mere bacterial challenge led to significant increases of both adhesion molecules' expressions compared to negative controls (11.72 ± 2.09 in ICAM-1, and 8.78 ± 0.95 in VCAM-1) and compared to β2 stimulation. These increases were even stronger under the combined administration of terbutaline and E. coli, the results were significant as compared to controls and to the β2 stimulation (30.91 ± 11.93 in ICAM-1, and 9.33 ± 1.13 in VCAM-1), but not when compared to bacterial stimulation.

## Discussion

Endothelial cells can attract and bind immunocompetent cells, and are involved in host defence. Stimuli like bacteria or ischemia can also induce an exaggerated expression and release of adhesion molecules [6,7] leading to a loss of shape and function of the endothelial cell layer, capillary leakage and development of interstitial oedema which may be either a cause or a consequence of microcirculation disorders. Clinical presentation of the systemic inflammatory response syndrome (SIRS) includes tachycardia, hypotension, fever, and respiratory insufficiency. Intensive care therapy is required in these patients to restore and maintain tissue perfusion and oxygenation. Treatment often remains limited to symptomatic therapy as the focus or other causes of this overwhelming reaction cannot definitely be found in many cases. Treatment includes volume replacement and vasopressor therapy for circulatory and microvascular resuscitation [5]. Glucocorticoids are often administered to reduce inflammatory activity, but have not been shown to reduce levels of soluble adhesion molecules in septic patients [8]. Adrenoceptor agonist drugs are almost regularly administered in the treatment of septic patients during intensive care therapy. Norepinephrine is given to maintain circulation in terms of increasing vascular resistance and organ perfusion. In case a patient suffers from bronchial obstruction, β2 agonists such as terbutaline can be given to improve ventilation.

Terbutaline as well as norepinephrine influence the properties of activated endothelial cells *in vitro *and *in vivo*: While norepinephrine led to an increase of interleukin-6 synthesis in LPS activated endothelial cells that was mediated by β1-receptors [9], terbutaline application affected cell adhesion in rats in terms of an enhanced leukocyte rolling on endothelial cells [10].

Endothelial adhesion molecules are crucial in mediating innate and acquired immunity. During general systemic inflammation, Law *et al. *[11] described a connection between multiple organ failure and increased levels of soluble ICAM-1 in peripheral blood of patients following multiple trauma in a group of 13 individuals. Sessler [12] also found increased levels of circulating ICAM-1 levels in patients with septic shock. Furthermore, ICAM-1 deficient mice were less prone to septic shock than mice without any genetic manipulation [13-15] which indicates the important role of ICAM-1 in conditions such as sepsis. Like Cowley *et al. *[16], Vargas Hein *et al. *[2] demonstrated that elevated sELAM-1 levels were corresponding with unfavourable outcomes of severe sepsis. sICAM-1 levels were not significantly different between survivors and non-survivors.

Carlson *et al. *showed in an *in vitro *experiment a reduced T-cell binding to activated endothelial cells after catecholamine application, this decrease however did not seem to be mediated by a change in adhesion molecule expression on either leukocytes or endothelial cells [17]. These effects may at least partly be mediated by a β1 and β2 induced increase of intracellular cAMP levels. Changes in transcription rates as a result of an increase of intracellular cAMP have been described for a variety of cells [18]. Investigation in the specific effects of increased cAMP levels on endothelial adhesion molecule expression did not lead to clear results. While Pober *et al. *found a reduced adhesion molecule expression after increasing cAMP levels in TNFα activated endothelial cells [19], other groups did not find effects of changes in cAMP concentrations on adhesion molecule expression [20,10]. Regarding the transcription level of adhesion molecules, expression is related to an increased activation of the transcription factor NF-κB [21]. Bacterial heat shock proteins also lead to an increased expression of cytokine and adhesion molecule expression in monocytes and endothelial cells [22].

As the interaction between adrenoceptor stimulation and adhesion molecule expression on stimulated endothelial cells has not been entirely clarified, our group investigated if β-adrenoceptor agonist administration caused an effect on expression of endothelial intercellular adhesion molecules (ICAM-1) and vascular adhesion molecules (VCAM-1) in human umbilical cord endothelial cells (HUVEC). Bacterial activation with *E. coli *itself caused significant increases in the expression of both adhesion molecules. Whereas β1 adrenoceptor stimulation with norepinephrine did not cause further increases, β2 stimulation with terbutaline led to a further increase in both ICAM-1 and VCAM-1 expressions.

Other groups have investigated different cell types such as pulmonary microvasculature cells [23] or different stimulation mechanisms and found evidence for migration of polymorphonuclear neutrophils mediated by endothelial adhesion molecules. Our method represents an isolated *in vitro *assay. Factors like underlying and concomitant diseases and their treatment are influencing adhesion molecule formation and release. We were interested if effects of other agents which are frequently used could be observed and lead to changed opinions and therapeutic consequences. In preliminary experiments we determined the optimal concentrations of stimulation and adrenoceptor treatment. Starting with either adrenoceptor or bacterial stimulation did not reveal different results.

We found an effect of bacterial stimulation on ICAM-1 and VCAM-1 adhesion molecule expression which was not markedly influenced neither by administration of the β1-adrenergic agent norepinephrine nor the β2-adrenergic agent terbutaline. In summary, adrenoceptor stimulation did not lead to further alterations. We have not examined soluble adhesion molecules which may represent a higher proportion of formerly expressed and then detached cells released into the assay or into the bloodstream as shown by other groups [6-8].

## Conclusions

Endothelial adhesion molecules ICAM-1 and VCAM-1 were increased following bacterial stimulation. Administration of adrenoceptor-stimulating substances did not lead to significant increases in stimulated or non-stimulated cells.

In forthcoming studies, the influence of adrenoceptor agonists on endothelial adhesion molecule release will be further investigated.

## Competing interests

H. R. received a grant from the local HiLF-Programme of Hannover Medical School for junior researchers which did not interfere with any possibly competing interests. The author(s) declare that they have no competing interests.

## Authors' contributions

HR has participated in the experimental parts of the study, and written the manuscript, BJ was involved in statistical analysis, MvG and BH have established the technique of HUVEC preparation and developed the experiments. WL and SW carried out and described the experiments. MP and DS have contributed to the manuscript. All authors have read and approved the manuscript.
